# Association of the sdLDL-C/lbLDL-C ratio with heart failure beyond conventional LDL-C levels: a cross-sectional study

**DOI:** 10.3389/fcvm.2026.1873514

**Published:** 2026-07-17

**Authors:** Xuyang Pan, Fang Liu, Wei Li, Tao Li, Changgao Zhou, Liting Yang, Yanchun Zhang, Xingyu Zhu, Lina Zheng, Yihan Zhou, Xue Sun, Shushen Ji, Yue Wang, Jiangman Zhao, Guoliang Yuan

**Affiliations:** 1 Department of Cardiovascular Medicine, ShuYang Hospital of Traditional Chinese Medicine, Shu Yang, Jiangsu, China; 2 Department of Internal Medicine, Kaifeng Central Hospital, Kaifeng, Henan, China; 3 Department of General Practice, Kaifeng Central Hospital, Kaifeng, Henan, China; 4 Shanghai Zhangjiang Institute of Medical Innovation, Shanghai Biotecan Pharmaceuticals Co., Ltd., Shanghai, China

**Keywords:** heart failure, lbLDL-C, left ventricular ejection fraction, lipid profile, sdLDL-C

## Abstract

**Background:**

Heart failure (HF) is one of the major cardiovascular diseases and the leading cause of death globally. Blood lipid profile is associated with HF early risk. Therefore, we aimed to explore the association between blood lipid profile and HF.

**Methods:**

A total of 584 newly diagnosed HF patients and 611 non-HF controls were included. Clinical characteristics and routine lipid parameters were collected. LDL-C subfractions (LDLC-1 to LDLC-7) were quantified using the LDL subfractions kit. Multivariable logistic regression analysis was performed to identify independent correlates of HF.

**Results:**

Among 584 HF patients and 611 controls, conventional LDL-C did not significantly differ between groups. However, HF patients had significantly higher sdLDL-C level, lower lbLDL-C level, and higher sdLDL-C/lbLDL-C ratio than controls. Multivariable logistic regression identified sdLDL-C/lbLDL-C ratio (OR = 2.199, 95%CI: 1.293–3.874, *P* < 0.001) as independent factor associated with HF.

**Conclusions:**

Lipoprotein subfraction characteristics, particularly the sdLDL-C/lbLDL-C ratio, is significantly associated with HF status and cardiac function.

## 1 Background

Heart failure (HF) is a major cause of hospitalization among individuals over 65 years old, and 21% of patients hospitalized for HF are readmitted within a month after discharge (1). HF is a complex clinical syndrome characterized either by the heart's inability to generate sufficient cardiac output to meet the body's metabolic demands or by the maintenance of an apparently adequate output only through compensatory neurohumoral stimulation (2). According to the differences in left ventricular ejection fraction (LVEF) and the changes after treatment, HF can be classified into four subtypes: HF with reduced ejection fraction (HFrEF), HF with improved ejection fraction (HFimpEF), HF with mildly reduced ejection fraction (HFmrEF), and HF with preserved ejection fraction (HFpEF) (3).

Dyslipidemia is a well-established risk factor for cardiovascular disease (CVD) and has also been implicated in the pathogenesis of HF (4). Experimental studies have shown that high-density lipoprotein cholesterol (HDL-C) exerts cardioprotective effects via antioxidant, anti-inflammatory, anti-apoptotic, and endothelial-protective properties (5). Studies have shown that higher levels of low-density lipoprotein cholesterol (LDL-C) increase the risk of both HFrEF and HFpEF subtypes (6). Oxidized LDL particles are particularly toxic because they stimulate the activity of macrophages and monocyte cytokines. These cytokines trigger an inflammatory cascade (7). However, these associations remain inconsistent across HF phenotypes, and LDL-C alone shows limited ability to capture residual cardiovascular risk or reflect the complexity of lipid-related pathophysiological processes in HF.

Emerging evidence indicates that LDL particles are highly heterogeneous in size, density, and metabolic behavior. Based on particle characteristics, LDL can be subclassified into large buoyant LDL (lbLDL) and small dense LDL (sdLDL) (8). Among these, sdLDL exhibits a markedly higher atherogenic potential due to its prolonged plasma half-life, reduced receptor-mediated clearance, enhanced arterial wall penetration, increased susceptibility to oxidative modification, and stronger binding affinity to proteoglycans within the vessel wall (9, 10). A growing body of evidence has demonstrated that elevated sdLDL-C is independently associated with coronary artery disease, carotid atherosclerosis, metabolic syndrome, diabetes mellitus, and adverse cardiovascular outcomes, even in individuals with normal or mildly elevated LDL-C levels (11, 12). Despite these findings, the role of LDL subfractions in HF remains insufficiently explored. Most previous studies focused on conventional lipid indices, while limited data were available regarding LDL subfraction profiles in HF populations or across HF phenotypes defined by left ventricular ejection fraction. Importantly, whether sdLDL-C and the balance between sdLDL-C and lbLDL-C are associated with HF presence and cardiac dysfunction remains unclear. In particular, the potential incremental value of LDL subfractions beyond traditional lipid measures has not been fully established in HF cohorts. This represents a critical gap in current knowledge, as it limits our understanding of lipid-related mechanisms in HF pathophysiology and restricts the development of more precise metabolic biomarkers for risk stratification.

Therefore, the present study aimed to comprehensively characterize LDL subfraction profiles in a cohort of patients with HF patients and non-HF controls. We further investigated the associations between LDL subfractions, HF status, and left ventricular systolic function. In particular, we focused on the sdLDL-C/lbLDL-C ratio as a potential integrated marker reflecting the lipid particle imbalance.

## 2 Methods

### 2.1 Study participants

This observational study enrolled 584 patients with HF and 611 non-HF controls who were admitted to the ShuYang Hospital of Traditional Chinese Medicine. Patients with were diagnosed with new-onset chronic HF in outpatient clinic according to the 2021 European Society of Cardiology (ESC) guidelines, and subsequently admitted for further evaluation and treatment (2). The diagnosis of HF was based on the presence of typical symptoms and signs, echocardiographic evidence of structural and/or functional cardiac abnormalities, and an elevated NT-proBNP level (>125 ng/L), in accordance with the ESC guidelines. Patients were classified as HF with reduced ejection fraction (HFrEF, LVEF ≤ 40%, *n* = 122), mildly reduced ejection fraction (HFmrEF, LVEF = 41%–49%, *n* = 96), or preserved ejection fraction (HFpEF, LVEF ≥ 50%, *n* = 366) (2) according to the ESC guidelines. The non-HF participants were individuals who underwent routine health examinations including transthoracic echocardiography. Non-HF controls were defined as no history of HF or other clinically diagnosed structural heart disease, and normal cardiac function confirmed by transthoracic echocardiography.

This study protocol was approved by the Ethics Committee of ShuYang Hospital of Traditional Chinese Medicine (20250804-001).

### 2.2 Data collection and study variables

Data on the following basic characteristics were collected: age, gender, body mass index (BMI), history of smoking, drinking status, preexisting disease including hypertension, coronary heart disease(CHD), and valvular heart disease. Laboratory parameters included serum creatinine (SCr), uric acid, total cholesterol (TC), triglyceride (TG), HDL-C, LDL-C, B-type natriuretic peptide (BNP), serum potassium, serum sodium, albumin, white blood cell (WBC) count, total lymphocyte, C-reactive protein(CRP), hematocrit, activated partial thromboplastin time (APTT), D-Dimer (D-D), cardiac troponin T (cTnT) and lipoprotein-associated phospholipase A2 (Lp-PLA2). Echocardiography results included left ventricular ejection fraction (LVEF), ratio of early to late mitral inflow velocity (E/A ratio), left ventricular end-diastolic diameter (LVEDD), left atrial diameter(LAD) and tricuspid regurgitation (TR).

### 2.3 Blood lipid profile detection

Blood samples were collected using serum separation tubes and anticoagulant tubes. Plasma was immediately separated after centrifugation at 800 × g for 10 min at 4 °C. TC, TG, HDL-C and LDL-C were measured in the clinical laboratory. The 2016 Chinese Guidelines for the Management of Dyslipidemia in Adults define the reference ranges for TC, TG, HDL-C, and LDL-C (8).

LDL-C subfractions were classified and quantified using the LDL subfraction kit from Shanghai Biotecan Pharmaceuticals Co., Ltd. In brief, plasma and liquid loading gel were added to the top of premade 3% polyacrylamide gel tubes. After photopolymerization at room temperature for 30 min, the samples were electrophoresed in an electrophoresis apparatus (Shanghai Biotecan Pharmaceuticals Co., Ltd.) for 70 min (3 mA per tube). Density measurements were then performed using a gel scanner (Hunan Biotecan Medical Devices Co., Ltd.). Finally, LDL-C was separated into seven subfractions (LDLC-1 to LDLC-7).

### 2.4 Statistical analysis

All statistical analyses were performed with GraphPad Prism (version 10.6; GraphPad Software, Inc.) and Statistical Package for Social Sciences (SPSS; IBM, NY, USA). Data on baseline characteristics were tabulated. Categorical variables were presented as counts and percentages. Continuous variables were presented as mean ± standard deviation (SD) of normally distributed data or median (interquartile range, IQR) for non-normally data. Continuous variables were compared using the Kruskal–Wallis test or one-way ANOVA followed by Bonferroni-adjusted *post hoc* tests to control for multiple pairwise comparisons., while discrete variables were compared using Fisher's exact test or chi-square test, as appropriate. Spearman's rank correlation analysis was used to evaluate linear correlations between continuous clinical and lipid variables.

Univariate logistic regression analysis was initially conducted to screen correlates of HF. Variables with a *P* value <0.05 in the univariate analysis or those considered clinically relevant based on previous evidence were entered into the multivariable logistic regression model. Age, sex, drinking status, hypertension, serum creatinine, and uric acid were included as adjustment covariates because they were well-recognized factors associated with HF or lipid metabolism. Prior to multivariable modeling, multicollinearity across lipid markers was evaluated via variance inflation factor (VIF) and tolerance values. Variables with a VIF > 10 or tolerance <0.10 were considered to indicate significant multicollinearity. LDLC-1 and LDLC-2 were excluded from the multivariable model to avoid inherent collinearity with lbLDL-C. Adjusted odds ratios (ORs) and 95% confidence intervals (CIs) were calculated to identify independent factors associated with HF. *P* < 0.05 was defined as statistically significant.

## 3 Results

### 3.1 Baseline characteristics

In total, 1195 participants were enrolled from the ShuYang Hospital of Traditional Chinese Medicine. The cohort consisted of 96 HFmrEF patients, 366 HFpEF patients, 122 HFrEF patients, and 611 non-HF control subjects. As displayed in Table 1, the majority of patients with HF were men (52.6%), and the proportion of male participants was significantly higher in patients with HF than in controls (34.7%), especially in the HFrEF group (59.8%). HF patients were significantly older than non-HF controls (*P* < 0.001). The proportions of participants with hypertension, CHD, and valvular heart disease in the HF group were higher than in the non-HF group. Additionally, we found elevated SCr levels and reduced concentrations of TC, TG, and HDL-C in the HF group.

**Table 1:** Baseline characteristics of non-HF controls and HF subtypes.

Characteristics	Non-HF (*n* = 611)	HF				*P* value
		HFrEF (*n* = 122)	HFmrEF (*n* = 96)	HFpEF (*n* = 366)	Total (*n* = 584)	
Age (years)	42 [37, 52]	73 [63, 80]	76 [69, 81]	70 [60, 79]	72 [63, 80]	<0.001
Male, *n* (%)	212 (34.7)	73 (59.8)	47 (49.0)	187 (51.1)	307 (52.6)	<0.001
BMI (kg/m^2^)	24.0 [22.0, 26.1]	24.1 [21.3, 26.0]	25.3 [22.2, 27.3]	24.6 [22.5, 26.8]	24.7 [22.0, 26.7]	0.009
Smoking, *n* (%)	61 (10.0)	22 (18.0)	15 (15.6)	79 (21.6)	116 (19.9)	0.103
Drinking, *n* (%)	86 (14.1)	11 (9.0)	7 (7.3)	38 (10.4)	56 (9.6)	<0.001
Hypertension, *n* (%)	18 (2.9)	56 (45.9)	53 (55.2)	231 (63.1)	340 (58.2)	<0.001
CHD, *n* (%)	0 (0.0)	57 (46.7)	46 (47.9)	130 (35.5)	233 (339.9)	<0.001
Valvular Heart Disease, *n* (%)	0 (0.0)	8 (6.8)	11 (11.7)	12 (3.3)	31 (5.3)	<0.001
SCr, mmol/L	61.0 [54.0, 73.0]	71.7 [58.0, 88.4]	70.0 [54.0, 92.5]	65.2 [52.9, 81.0]	67 [54, 85.7]	<0.001
Uric acid, mg/dL	284.0 [236.5, 349.5]	325.0 [268.6, 424.0]	318.4 ± 131.3	283.3 [218.0, 365.0]	309 [240.9, 399.7]	<0.001
TC, mmol/L	4.5 [4.0, 5.1]	3.6 [3.1, 4.4]	3.9 ± 1.2	4.2 [3.4, 5.1]	3.8 [3.0, 4.6]	<0.001
TG, mmol/L	1.2 [0.9, 1.9]	1.0 [0.8, 1.4]	1.1 [0.8, 1.4]	1.3 [0.9, 2.2]	1.15 [0.9, 1.7]	<0.001
HDL-C, mmol/L	1.2 [1.1, 1.4]	0.9 [0.2, 1.1]	0.9 [0.4, 1.2]	0.9[0.4, 1.2]	0.9 [0.4, 1.2]	<0.001
LDL-C, mmol/L	2.5 [2.1, 3.0]	2.4 [1.7, 3.4]	2.2 [1.7, 3.0]	2.4 [1.8, 3.4]	2.4 [1.8, 3.3]	0.06
LVEF (%)	/	36 [30, 39]	45 [43, 47]	58 [55, 62]	47 [40, 56]	<0.001
BNP (mg/dL)	/	4,155.5 [2,305.0, 8,256.5]	2,765.0 [1,033.4, 5,084.8]	704.8 [141.7, 2,035.0]	2,290.0 [685.1, 5,128]	<0.001
NYHA class III/IV, *n* (%)	/	107 (91.5)	78 (83.9)	120 (71.0)	305 (52.2)	<0.001
E/A ratio <1 (%)	/	88 (75.9)	70 (76.9)	308 (88.8)	466 (79.8)	0.001
LVEDD, mm	/	56.0 [49.0, 61.0]	48.0 [46.0, 53.0]	46.0 [45.0, 48.0]	49.0 [45.0, 55.0]	<0.001
LAD, mm	/	45.0 [41.0, 48.3]	44.0 [41.0, 47.0]	37.0 [35.0, 42.0]	44.0 [37.0, 47.0]	<0.001
TR, *n* (%)						<0.001
None	/	/	5 (5.2)	144 (39.7)	149 (25.5)	
Mild	/	5 (4.1)	55 (57.3)	159 (43.8)	219 (37.5)	
Moderate	/	49 (40.2)	26 (27.1)	48 (13.2)	123 (21.1)	
Severe	/	52 (42.6)	10 (10.4)	12 (3.3)	74 (12.7)	
K (mmol/L)	/	4.0 [3.7, 4.2]	4.0 [3.6, 4.4]	3.9 [3.7, 4.2]	4.0 [3.6, 4.3]	0.875
Na (mmol/L)	/	137.8 [134.7, 140.2]	138.5 [136.0, 140.2]	138.8 [136.6, 141.0]	139.5 [136.9, 141.2]	0.006
Albumin	/	39.5 [36.4, 42.7]	40.1 [37.0, 42.8]	42.2 [39.1, 44.8]	40.2 [37.9, 42.7]	<0.001
WBC count, 10^9^/L	/	6.0 [4.7, 7.4]	6.3 [4.6, 7.5]	6.3 [5.3, 7.9]	6.3 [4.8, 7.5]	0.178
Total lymphocyte, 10^9^/L	/	1.2 [0.8, 1.7]	1.2 [0.9, 1.6]	1.4 [1.1, 1.7]	1.4 [1.0, 1.6]	0.048
CRP	/	5.5 [2.0, 15.6]	5.7 [1.4, 17.7]	2.0 [0.8, 5.8]	2.8 [0.9, 10.6]	<0.001
Hematocrit	/	39.1 [35.9, 43.7]	39.4 [35.9, 44.0]	40.1 [35.9, 43.0]	38.0 [34.7, 41.3]	0.888
PT, s	/	13.4 [12.2, 15.2]	13.4 [12.4, 15.8]	12.1 [11.3, 13.3]	13.2 [12.2, 14.3]	<0.001
APTT, s	/	29.8 [26.6, 33.1]	30.1 [26.7, 35.6]	27.4 [25.2, 30.6]	29.0 [26.3, 33.0]	<0.001
D-D, mg/L	/	0.4 [0.2, 0.7]	0.4 [0.3, 0.5]	0.3 [0.2, 0.4]	0.3 [0.2, 0.6]	<0.001
cTnT, mg/d L	/	1.5 [0.0, 3.8]	1.3 [0.0, 2.8]	1.7 [0.0, 7.0]	1.7 [0.5, 5.3]	0.172
Lp-PLA2, ng/mL	/	132.4 [89.1, 204.6]	103.6 [64.4, 153.8]	25.4 [20.9, 52.2]	88.7 [45.4, 143]	<0.001

Data are presented as median [interquartile range (IQR)], or number (%). Continuous variables were compared using Kruskal–Wallis test. Categorical variables were compared using the chi-square test or Fisher's exact test, as appropriate.

HF, heart failure; HFrEF, heart failure with reduced ejection fraction; HFmrEF, heart failure with mildly reduced ejection fraction; HFpEF, heart failure with preserved ejection fraction; BMI, body mass index; CHD, coronary heart disease; SCr, serum creatinine; TC, total cholesterol; TG, triglycerides; HDL-C, high-density lipoprotein cholesterol; LDL-C, low-density lipoprotein cholesterol; LVEF, left ventricular ejection fraction; BNP, B-type natriuretic peptide; NYHA, New York Heart Association; E/A ratio, ratio of early (E) to late (A) mitral inflow velocity; LVEDD, left ventricular end-diastolic diameter; LAD, left atrial diameter; TR, tricuspid regurgitation; WBC, white blood cell; CRP, C-reactive protein; PT, prothrombin time; APTT, activated partial thromboplastin time; D-D, D-dimer; cTnT, cardiac troponin T; Lp-PLA2, lipoprotein-associated phospholipase A2.

### 3.2 Blood lipid profile in the non-HF controls and HF subtypes

We observed no statistically significant differences in LDL-C levels among the four groups (*P* = 0.06), but abnormal ratios of total LDL-C showed a statistically significant difference (*P* = 0.001). Less than 25% of HF patients exhibited elevated total LDL-C, confirming that most HF cases presented with conventionally normal LDL-C concentrations. HDL-C level was significantly lower in HF patients than in non-HF controls.

**Figure 1:**
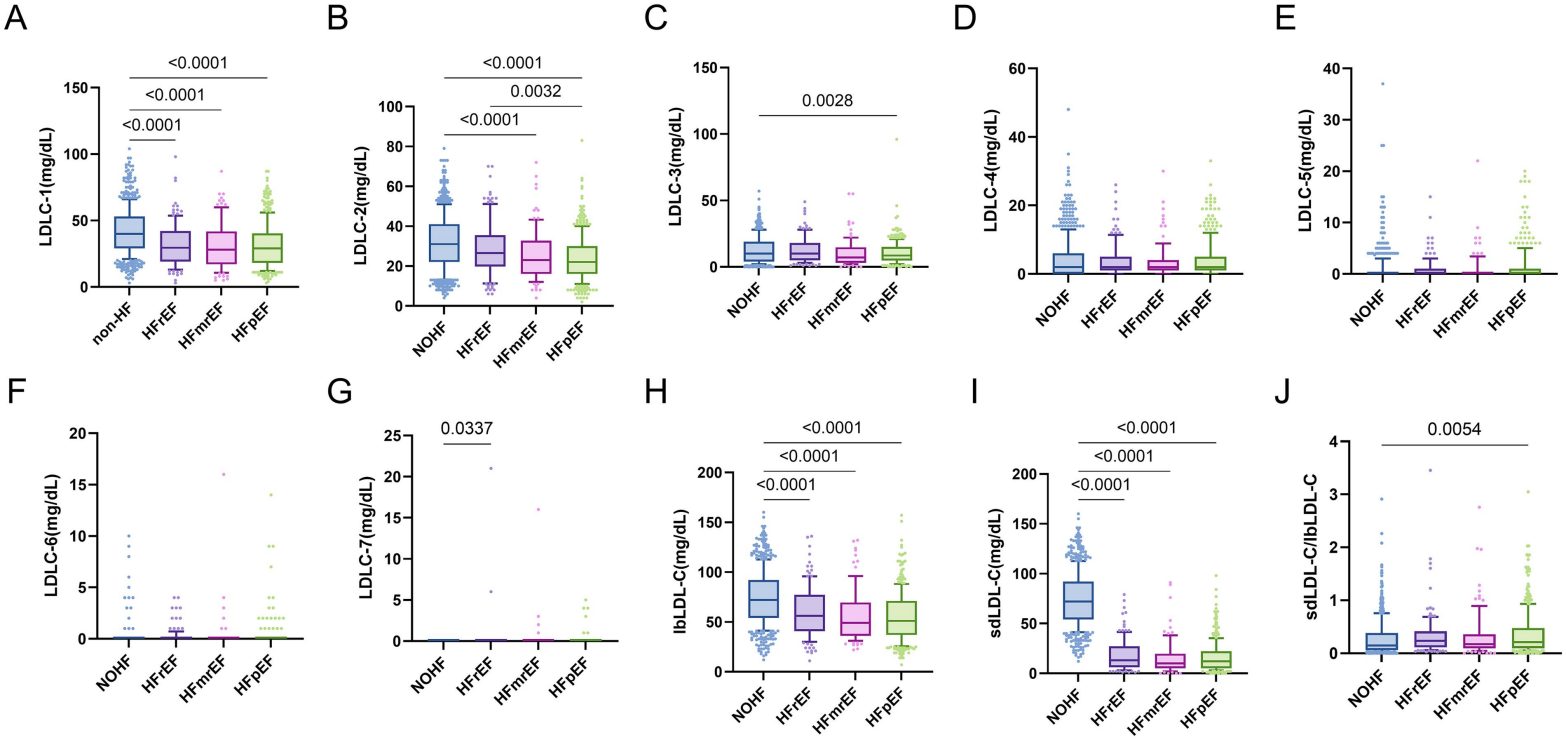
Comparisons of low-density lipoprotein cholesterol (LDL-C) subfraction profiles between heart failure with mildly reduced ejection fraction (HFmrEF), heart failure with reduced ejection fraction (HFrEF), heart failure with preserved ejection fraction (HFpEF), and Non-HF controls. The different levels of **(A)** LDLC-1, **(B)** LDLC-2, **(C)** LDLC-3, **(D)** LDLC-4, **(E)** LDLC-5, **(F)** LDLC-6, **(G)** LDLC-7, **(H)** sdLDL-C/lbLDL-C, **(I)** small dense low-density lipoprotein cholesterol (sdLDL-C), and **(J)** large buoyant low-density lipoprotein cholesterol (lbLDL-C) among four groups. Statistical comparisons among the four groups were performed using the Kruskal–Wallis test followed by Bonferroni-adjusted *post hoc* analyses.

To further analyze the blood lipid profile, we tested seven LDL-C subtype levels, and the results are shown in Table 2 and Figure 1. We found that LDLC-1, LDLC-2, and lbLDL-C were significantly lower in HF patients (*P* < 0.05) than in non-HF controls, but no significant difference was observed for LDLC-1 and lbLDL-C across the three HF subgroups. LDLC-2 levels were significantly reduced in HFmrEF and HFpEF patients vs. controls, while no meaningful LDLC-2 decline was detected in HFrEF subjects. SdLDL-C concentrations and the sdLDL-C/lbLDL-C ratio were significantly elevated in HF patients relative to controls.

**Table 2:** Comparisons of LDL subfractions profiles between HFmrEF, HFrEF, HFpEF, and Non- HF controls.

Characteristics	Non-HF (*n* = 611)		HF		*P* value
		HFrEF (*n* = 122)	HFmrEF (*n* = 96)	HFpEF (*n* = 366)	
LDL-C, mmol/L	2.5 [2.1, 3.0]	2.4 [1.7, 3.4]	2.2 [1.7, 3.0]	2.4 [1.8, 3.4]	0.06
LDLC-1, mg/dL	40.0 [29.0, 53.0]	29.5 [19.0, 42.0]	28 [17.0, 41.8]	30.0 [18.0, 40.3]	<0.001
LDLC-2, mg/dL	31.0 [22.0, 41.0]	26.5 [19.8, 35.5]	23 [16.0, 32.8]	22.0 [16.0, 30.0]	<0.001
LDLC-3, mg/dL	10.0 [4.0, 19]	10.0 [5.0, 18.0]	7.0 [3.0, 14.8]	8.5 [4.8, 15.0]	0.002
LDLC-4, mg/dL	2.0 [0.0, 6.0]	2.0 [1.0, 5.0]	2.0 [1.0, 4.0]	2.0 [1.0, 5.0]	0.90
LDLC-5, mg/dL	0.0 [0.0, 0.0]	0.0 [0.0, 1.0]	0.0 [0.0, 0.0]	0.0 [0.0, 1.0]	0.26
LDLC-6, mg/dL	0.0 [0.0, 0.0]	0.0 [0.0, 0.0]	0.0 [0.0, 0.0]	0.0 [0.0, 0.0]	0.204
LDLC-7, mg/dL	0.0 [0.0, 0.0]	0.0 [0.0, 0.0]	0.0 [0.0, 0.0]	0.0 [0.0, 0.0]	0.007
sdLDL-C, mg/dL	11.0 [4.0, 27.0]	13.0 [6.0, 27.0]	10.0 [5.0, 19.8]	11.0 [5.0, 22.0]	<0.001
lbLDL-C, mg/dL	72.0 [55.0, 92.0]	58.9 ± 26.5	49.0 [36.0, 69.5]	51.0 [37.0, 71.0]	<0.001
sdLDL-C/lbLDL-C	0.15 [0.06, 0.38]	0.23 [0.11, 0.42]	0.17 [0.09, 0.36]	0.21 [0.09, 0.47]	0.008

Data are presented as median (interquartile range [IQR]). Differences among groups were assessed using the Kruskal–Wallis test.

LDL-C, low-density lipoprotein cholesterol; LDLC-1 to LDLC-7, LDL cholesterol subfractions 1–7; lbLDL-C, large buoyant low-density lipoprotein cholesterol; sdLDL-C, small dense low-density lipoprotein cholesterol; HFrEF, heart failure with reduced ejection fraction; HFmrEF, heart failure with mildly reduced ejection fraction; HFpEF, heart failure with preserved ejection fraction.

### 3.3 Correlation analysis among HF status, clinical characteristics, and blood lipid profile

In Table 3, Spearman correlation analysis showed that TC (*r* = −0.233, *P* < 0.001), HDL-C (*r* = −0.386, *P* < 0.001), LDLC-1 (*r* = −0.314, *P* < 0.001), LDLC-2 (*r* = −0.258, *P* < 0.001), LDLC-3 (*r* = −0.071, *P* = 0.015), and lbLDL-C (*r* = −0.333, *P* < 0.001) were significantly negatively correlated with the presence of HF. In contrast, LDLC-4 (*r* = 0.108, *P* < 0.001), LDLC-5 (*r* = 0.086, *P* = 0.003), LDLC-6 (*r* = 0.105, *P* < 0.001), LDLC-7 (*r* = 0.103, *P* < 0.001), and the sdLDL-C/lbLDL-C ratio (*r* = 0.132, *P* < 0.001) showed a weak but statistically significant positive correlation with HF. No significant correlations with HF were observed for TG (*r* = 0.008, *P* = 0.786), LDL-C (*r* = –0.055, *P* = 0.055), and sdLDL-C (*r* = –0.003, *P* = 0.916).

**Table 3:** Correlation analysis of HF with blood lipid profile.

Variable	R value	*P* value
TG, mmol/L	0.008	0.786
TC, mmol/L	−0.233	<0.001
HDL-C, mmol/L	−0.386	<0.001
LDL-C, mmol/L	−0.055	0.055
LDLC-1, mg/dL	-0.314	<0.001
LDLC-2, mg/dL	−0.258	<0.001
LDLC-3, mg/dL	−0.071	0.015
LDLC-4, mg/dL	0.108	<0.001
LDLC-5, mg/dL	0.086	0.003
LDLC-6, mg/dL	0.105	<0.001
LDLC-7, mg/dL	0.103	<0.001
sdLDL-C, mg/dL	−0.003	0.916
lbLDL-C, mg/dL	−0.333	<0.001
sdLDL-C/lbLDL-C	0.132	<0.001

Data are presented as Spearman's correlation coefficients (*r* values) with corresponding *P* values. *P* values <0.05 were considered statistically significant.

HF, heart failure; TG, triglyceride; TC, total cholesterol; HDL-C, high-density lipoprotein cholesterol; LDL-C, low-density lipoprotein cholesterol; LDLC-1 to LDLC-7, low-density lipoprotein cholesterol subfractions 1 to 7; sdLDL-C, small dense low-density lipoprotein cholesterol; lbLDL-C, large buoyant low-density lipoprotein cholesterol.

### 3.4 Lipids associated with HF

Multivariable logistic regression analysis was performed after adjustment for age, sex, drinking status, hypertension, serum creatinine, and uric acid. As shown in Table 4, TC (OR = 0.648, 95% CI: 0.490–0.857, *P* = 0.002), HDL-C (OR = 0.159, 95% CI: 0.086–0.294, *P* < 0.001), and LDLC-3 (OR = 0.956, 95% CI: 0.922–0.992, *P* = 0.016) were independently associated with lower odds of HF, whereas the sdLDL-C/lbLDL-C ratio was independently associated with higher odds of HF (OR = 2.199, 95% CI: 1.293–3.874, *P* < 0.001).

**Table 4:** Odds ratios for factors for HF from univariate and multivariate stepwise logistic regression analyses.

Varibles	Univariate analysis		Multivariate analysis		Multicollinearity analysis	
	OR(95%CI)	*P* value	OR(95%CI)	*P* value	VIF	Tolerance
TG	0.977 (0.928–1.028)	0.367				
TC	0.698 (0.625–0.780)	<0.001	0.648 (0.490–0.857)	0.002	1.479	0.676
HDL-C	0.150 (0.106–0.214)	<0.001	0.159 (0.086–0.294)	<0.001	1.147	0.872
LDL-C	1.140 (1.044–1.245)	0.003	1.734 (1.289–2.332)	<0.001	1.016	0.984
LDLC-1	0.965 (0.958–0.972)	<0.001				
LDLC-2	0.962 (0.953–0.971)	<0.001				
LDLC-3	0.981 (0.969–0.993)	0.002	0.956 (0.922–0.992)	0.016	2.116	0.473
LDLC-4	0.998 (0.978–1.017)	0.830				
LDLC-5	1.031 (0.991–1.073)	0.135				
LDLC-6	1.142 (0.996–1.308)	0.057				
sdLDL-C	0.995 (0.988–1.002)	0.152				
lbLDL-C	0.975 (0.970–0.979)	<0.001	0.989 (0.977–1.002)	0.096	2.284	0.438
sdLDL-C/lbLDL-C	1.671 (1.226–2.277)	0.001	2.199 (1.293–3.874)	<0.001	2.475	0.404

Multivariable model adjusted for age, sex, drinking status, hypertension, serum creatinine, and uric acid. Variables with *P* < 0.05 in univariable analysis were entered into the multivariable model. *P* values <0.05 were considered statistically significant.

HF, heart failure; OR, odds ratio; CI, confidence interval; TG, triglyceride; TC, total cholesterol; HDL-C, high-density lipoprotein cholesterol; LDL-C, low-density lipoprotein cholesterol; LDLC-1 to LDLC-7, low-density lipoprotein cholesterol subfractions 1 to 7; sdLDL-C, small dense low-density lipoprotein cholesterol; lbLDL-C, large buoyant low-density lipoprotein cholesterol, VIF: variance inflation factor.

## 4 Discussion

Over the past several decades, numerous studies have reported that many clinical variables, such as hypertension (13), blood lipid (14), and gender (15), are closely associated with the risk of HF. HF with reduced ejection fraction was a major contributor to cardiovascular morbidity and mortality (16). Thus, in this study, we recruited 611 non-HF controls and 584 HF patients to identify the potential factors associated with HF and reduced LVEF. We found that patients with different types of HF exhibited partially distinct lipid profiles. Our findings suggest that LDL subfraction profiles, particularly the sdLDL-C/lbLDL-C ratio, is significantly associated with HF status, warranting further prospective investigation to explore their potential role in risk stratification.

Firstly, we found that age and hypertension are independently associated factors. Consistent with our results, a prior cohort of 5,143 individuals reported hypertension as the antecedent condition in 91% of new-onset HF cases (17). Cardiac adaptation to persistent pressure overload was characterized by diastolic dysfunction and concentric hypertrophy of the left ventricle. With continued elevation of pressure, diastolic impairment worsened, ventricular filling of the remodeled left ventricle declined, and HFpEF developed. Among hypertensive patients, diastolic dysfunction and HFpEF represented the most prevalent forms of cardiac involvement (18).

It is well known that the lipid profile is closely associated with cardiovascular diseases. Similar to the other study (19), we also observed that patients with HF exhibited a lipid profile different from that of healthy individuals. Multiple studies have demonstrated that, beyond its established contribution to vascular atherosclerosis, hyperlipidemia can exert direct detrimental effects on the myocardium, resulting in greater susceptibility to ischemia/reperfusion injury and a diminished efficacy of cardioprotective strategies such as ischemic preconditioning and postconditioning (20). In the absence of significant coronary artery narrowing, chronic hyperlipidemia promoted lipid accumulation within the myocardium, thereby impaired both cardiac performance and electrophysiological function (21).

In addition, we found no significant difference in LDL-C level in the HF patients. Therefore, we further detected the concentrations of LDL-C subfractions. Investigation of sdLDL-C and lbLDL-C may offer further advantages because these could provide additional insights into lipid abnormalities in HF. Surprisingly, our study showed that patients in the HFrEF, HFpEF and HFmrEF groups experienced varying degrees of lbLDL-C reduction, with both LDLC-1 and LDLC-2 showing a downward trend. The level of sdLDL-C also increased among different HF subtypes groups. Moreover, we found that different LDL-C subtypes exhibit varying correlations with the occurrence of HF, BNP levels, NYHA classification and LVEF levels, indicating that focusing on specific LDL-C subtypes is more meaningful than merely considering the relationship between LDL and disease. In a longitudinal study conducted in 2025, the researchers found a significant association between sdLDL-C and coronary calcium scan (CAC) incidence, and lbLDL-C concentrations were inversely associated with CAC progression (22). Our findings suggest that further investigation of LDL subfractions may help refine risk stratification, although the cross-sectional design precludes causal inferences.

Accumulating evidence has shown that sdLDL-C is a robust factor in various diseases by showing predictive power even in individuals with optimal LDL-cholesterol levels (<100 mg/dL). Lee et al. reported that sdLDL-C was associated with insulin resistance in subjects with early-stage impaired glucose metabolism (23). St-Pierre et al. found that sdLDL-C particles, particularly in subjects with insulin resistance, is closely associated with cardiovascular disease(CVD) and are recognized as a surrogate marker for CVD (24). SdLDL-C particles exhibit a higher propensity for uptake by arterial tissues compared with lbLDL-C particles, indicating enhanced transendothelial transport of the smaller particles. Additionally, sdLDL-C particles demonstrate reduced receptor-mediated clearance, increased affinity for arterial wall proteoglycans, and greater susceptibility to oxidative modification (25).

Beyond its well-established pro-atherogenic properties, sdLDL-C may also contribute to HF pathophysiology through interactions with the coagulation and fibrinolytic systems. Patients with HF commonly exhibited a prothrombotic state characterized by coagulation pathway activation, impaired fibrinolysis, endothelial dysfunction, and systemic inflammation, all of which are associated with adverse cardiovascular outcomes (26, 27). Small dense LDL concentration correlated independently with plasminogen activator inhibitor-1 (PAI-1) activity, suppressing fibrinolysis and fostering a prothrombotic milieu that may exacerbate myocardial ischemia and adverse cardiac remodeling in HF (28). However, direct evidence linking sdLDL subfractions to specific hemostatic biomarkers in HF populations remained limited, and our cross-sectional study was not designed to assess these mechanistic pathways. Future prospective studies integrating comprehensive lipid subfraction profiling with hemostatic biomarker panels are warranted to determine whether the sdLDL-C/lbLDL-C ratio reflects not only an atherogenic lipid imbalance but also an underlying prothrombotic state in HF (29).

An alternative interpretation for our findings was that the observed alterations in lipid profiles may represent a consequence rather than a cause of HF. Chronic HF was associated with a constellation of metabolic derangements, including hepatic congestion, reduced cardiac output leading to decreased hepatic perfusion, and systemic inflammation, all of which may impair hepatic lipoprotein synthesis and clearance (30–32). Consequently, the altered distribution of LDL subfractions observed in the present study may reflect metabolic remodeling secondary to HF. Therefore, given the retrospective cross-sectional design of the present study, our findings should be interpreted as demonstrating an association rather than causation. Whether the observed lipid subfraction alterations precede HF onset or occur as a consequence of cardiac dysfunction remains to be determined in future longitudinal studies with repeated lipid profiling and detailed clinical phenotyping.

Several limitations should be acknowledged. First, the cross-sectional design precludes causal inference, and although we adjusted for multiple potential confounders, residual confounding from unmeasured factors (e.g., detailed past medical history, medication use, particularly lipid-lowering therapies) could not be excluded. Second, hospital-based recruitment may introduce selection bias by over-representing patients with more severe disease, and the exclusion of patients who died before discharge may have introduced survivor bias, potentially underestimating the true associations. Third, this was a single-center study conducted exclusively in a Chinese population, limiting the external generalizability of our findings. Despite these limitations, our study has notable strengths, including a large sample size, comprehensive lipid subfraction profiling, and detailed clinical characterization, providing a foundation for future multi-center prospective validation.

## 5 Conclusion

In summary, compared with conventional lipid profiles, low-density lipoprotein subfractions showed statistically significant but modest associations with reduced ejection fraction in HF patients. A comprehensive assessment of lipoprotein subfractions may improve the characterization of lipid alterations in HF. However, prospective studies are required to determine their potential role in disease development and risk stratification.

## Data Availability

The original contributions presented in the study are included in the article/Supplementary Material, further inquiries can be directed to the corresponding authors.
